# Treatment of COVID-19: Perspective on Convalescent Plasma Transfusion

**DOI:** 10.3389/fmed.2020.00435

**Published:** 2020-07-28

**Authors:** Ryan M. Farhat, Mohammad A. Mousa, Eshaan J. Daas, Marilyn K. Glassberg

**Affiliations:** ^1^College of Medicine Phoenix, University of Arizona, Phoenix, AZ, United States; ^2^Division of Pulmonary Critical Care and Sleep Medicine, Department of Medicine, University of Arizona College of Medicine-Phoenix, Phoenix, AZ, United States

**Keywords:** coronavirus (2019-nCoV), SARS–CoV-2, transfusion safety, neutralizing antibody titers (NAT), convalescent plasma for COVID-19 therapy

## Abstract

The novel coronavirus (COVID-19) has continued its global spread since the first documented case in late 2019 in Wuhan, China. With over 10 million cases and 500 thousand deaths reported worldwide, the need for an effective treatment regimen is evident. Historically, convalescent plasma (CP) has been utilized in the treatment of viral respiratory pathogens. Critically ill patients with COVID-19 in China and South Korea have been treated with CP given the ineffectiveness of experimental therapies with antivirals alone. This commentary explores the importance of published experience and the pending establishment of efficacy to facilitate an informed decision regarding the therapeutic use of CP. With increasing mortality around the world from COVID-19 infection, the need for alternative, effective treatment regimens is critical.

## The Novel Coronavirus Outbreak

As of June 29, 2020, there have been over 10 million reported cases and over 500,000 deaths from the COVID-19 infection (1). Although many clinical trials are in progress, there is a lack of data supporting any treatments including favipiravir, remdesivir, hydroxychloroquine, and lopinavir (2–4). Historically, convalescent plasma (CP) has been utilized in the treatment of viral respiratory pathogens (5). Patients have undergone transfusion of pathogen specific antibodies from donors who recovered from the virus, providing them with passive immunization (6–9). With this historical perspective, critically ill patients with COVID-19 in China and South Korea have been treated with CP given the ineffectiveness of experimental therapies with antivirals alone (10–14). There are ongoing randomized, double-blind trials, with one study showing insignificant improvement after 28 days using CP along with standard treatment, although the study was determined to be underpowered and terminated early (15). As of June 29, 2020, the Food and Drug Administration (FDA) has not approved the use of CP for use in COVID-19 patients. This commentary explores the importance of published experience and the pending establishment of efficacy to facilitate an informed decision regarding the therapeutic use of CP. With increasing mortality around the world from COVID-19 infection, the need for alternative, effective treatment regimens is critical.

## Advantages of CP Against SARS-CoV-2

Studies using CP during SARS, influenza pandemic, and MERS demonstrate potential for clinical efficacy during the COVID-19 pandemic. A meta-analysis of 1,700 patients during the 1918 Spanish influenza revealed a 16% fatality rate in patients treated with CP compared to 37% in those not treated with CP. Mortality was 20% in patients treated with CP compared to 54.8% in those not treated with CP in a study in patients with severe H1N1. Patients in the treatment group had a lower viral load on days 3, 5, and 7 after transfusion and had lower levels of inflammatory cytokines such as IL6, IL10, and TNF (16). Another study in patients with H1N1 also reported similar clinical outcomes (17). Shorter hospital stays and lower mortality were found in patients with SARS who received CP than patients treated with methylprednisolone alone (6). Patients treated with neutralizing antibody achieving titers (NAT) of at least 1:80 during the MERS outbreak showed clinical improvement compared to lower titers (18). There were no adverse effects noted in the aforementioned studies.

Due to these promising results in patients with SARS and H1N1 infections, CP has been studied as a treatment option in COVID-19 (10–15) (Table 1). The titers of CP are based on recommendations from the FDA of at least 1:160 or 1:80 when a more concentrated alternative is unavailable (19). Five critically ill patients with COVID-19-related pneumonia who failed corticosteroids and antiviral treatments alone were given 2 doses of between 200 and 250 mL of CP therapy between days 10 and 22. The NAT of the donors were <40. Viral loads became undetectable, pulmonary infiltrates decreased by day 3, and CRP and procalcitonin levels also decreased (10). In a separate study, ten patients with severe COVID-19 were treated with 200 mL of CP after an average of 16.5 days after symptom onset. The NAT of the donors was 1:640. Seven patients showed improved oxyhemoglobin saturation, decreased CRP, improved lymphocyte counts, and undetectable viral load after transfusion (11). Patients treated as late as 4 weeks after symptom onset with 200 mL of CP in Wuhan, China, showed clinical and radiological improvement, although NAT were unknown (12). A single report demonstrated that mechanical ventilation was not required 11 days after a transfusion containing titers >1:320 of IgG to COVID-19 (14).

**Table 1:** Summary of recently published studies of convalscent plasma transfusion in COVID-19 patients.

	**Shen (*N* = 5) (10)**	**Duan (*N* = 10) (11)**	**Ye (*N* = 6) (12)**	**Ahn (*N* = 2) (13)**	**Zhang (*N* = 1) (14)**	**Li (*N* = 103) (15)**
CP administration^*^	10–22 days after admission	Average 16.5 days after symptom onset	At least 4 weeks after symptom onset	Day 7 for patient 1 and day 22 for patient 2 after symptom onset	Day 17 after admission	Median interval was 30 days after symptom onset
Dose of CP	2 consecutive transfusions of 200 - 250 mL	One dose of 200 mL	At least one dose of 200 mL	Two doses of 250 mL	One dose of 200 mL	Median infusion was 200 mL
Donor Neutralizing Antibody Titer	ELISA antibody titer > 1:1000, end point dilution titer > 40	> 1:640	Not reported	Not analyzed	> 1:320	>1:640
Results	- No documented adverse effects - All 5 patients had a decrease in viral load within 12 days of transfusion and improved clinically	- No serious adverse reactions except minor facial rash in one patient - Viral load undetectable in 7 patients after 7 days	- No documented adverse effects - Persistent positive throat swab tests and no increase in titers in Patient #1 after three rounds of CP therapy - Positive clinical and radiographical impact reported - Unclear if benefit from CP or natural course of virus	- No documented adverse effects - Clinical and radiographical improvement - Decreased viral load after transfusion - Unclear if benefit from CP or natural course of virus	- No documented adverse effects - Decreased lymphocyte count after transfusion - Patient removed off mechanical ventilation 11 days after transfusion	- One definite nonsevere allergic transfusion reaction and one possible severe transfusion associated dyspnea - No statistical significance in clinical improvement prior to termination - Study was terminated early due to decreased patient recruitement as a result of virus containment

* *All patients recieved concurrent administration of antivirals and steroids*.

## Limitations of CP Implementation

There are several significant concerns and disadvantages of CP in the treatment of patients with COVID-19. The transfusion of blood products, specifically plasma, is associated with a few rare adverse effects. Severe effects include transfusion-related acute lung injury (TRALI), transfusion-associated circulatory overload (TACO), and anaphylactic reactions. Minor effects include transmission of disease and hemolytic and non-hemolytic transfusion reactions (20). Two cases of patients with Ebola virus and MERS-CoV who received CP developed acute respiratory distress syndrome (ARDS) thought to be a result of TRALI (21, 22). Pathogen reduction technology during the apheresis of CP has been demonstrated to mitigate viral activity in blood products. Currently, the efficacy of inactivating SARS-CoV-2 in collected plasma using similar technology has not been tested (5).

There are also limitations in the use of CP not directly associated with clinical outcomes. Donor eligibility requirements account for factors such as age, sex, weight, and symptom course. Specifically, plasma donors in the United States must pass a medical examination and be 18 years of age weighing at least 50 kg (23). Additional pre-donation requirements include assessment of microbiology and anti-HLA Ab. The current recommended timeline for CP use calls for plasma donation 14 to 28 days following symptom resolution^5^ coupled with the recommendation to initiate treatment by day 5 at the latest to improve survival, prevent clinical deterioration, and shorten hospitalization (24). The NAT threshold in over one hundred COVID-19 CP investigations ranges between 1:160 and 1:640, presenting an additional consideration prior to transfusion. Treatment includes both antiviral and CP use, rendering it difficult to identify the individual effects of CP therapy (11). The possibility of negative clinical outcomes and limitations to implementation combined with the lack of randomized controlled trials (RCT) constitute the potential downsides of CP treatment in patients with COVID-19 infection.

## Interpretation and Recommendation

The lack of a successful treatment for SARS-CoV-2 coupled with a devastating number of cases supports the consideration of CP as a treatment. Reviews of recently published studies and historical use of CP suggest that convalescent plasma can be considered as an experimental treatment in critically ill patients as supported by the FDA (19). A recent open-label RCT showed no statistically significant effect of CP (1:160) in addition to standard treatment on mortality at 28 days post treatment or time to discharge (15). Limitations of this study included early termination, a small sample size, and lack of standardization of methods and procedures. A recent review underscores the lack of randomized, double-blind trials and highlights the importance of considering a “scale up” study in order to utilize CP once efficacy is established (25).

There are many parameters of plasma collection and transfusion that must be considered to maintain the safety of both donors and patients. Current national requirements for plasma donation in the United States should be followed including age and weight restrictions. Plasma should be collected from confirmed positive patients who are asymptomatic for between 10 and 28 days and subsequently test negative for SARS-CoV-2 and other viral illnesses. Treatment should be initiated by Day 5 of symptomatic presentation in critically ill patients to decrease length of hospital stay and mortality. NAT threshold should follow the FDA recommendation of 1:160 or 1:80 *in situ*ations with no alternatives (19). Improvements in diagnosing patients with COVID-19 has resulted in greater identification of those with the disease, which can help aid a greater number of patients with CP transfusion (26).

With total mortality still on the rise, convalescent plasma should be considered as an experimental therapeutic approach. The published cases suggest positive clinical outcomes in patients receiving CP in addition to antiviral therapy +/– corticosteroids (27). The increasing availability of reliable testing worldwide should facilitate the identification of potential donors. Additional studies to determine titers and establish long-term clinical outcomes are needed to confirm any potential benefit of CP therapy.

CP shows promise as an effective therapeutic option for patients with COVID-19. There are currently multiple randomized trials in progress purportedly measuring the therapeutic benefit of CP (clinicaltrials.gov, accessed June 29, 2020). Determination of “responders” and “non-responders” in robustly conducted clinical trials will establish optimal dosing and efficacy and support the use of CP as standard treatment instead of experimental therapy for COVID-19 infection.

## Data Availability Statement

All datasets presented in this study are included in the article/supplementary material.

## Author Contributions

All authors listed have made a substantial, direct and intellectual contribution to the work, and approved it for publication.

## Conflict of Interest

The authors declare that the research was conducted in the absence of any commercial or financial relationships that could be construed as a potential conflict of interest.
